# Detrimental Effects of Online Pro–Eating Disorder Communities on Weight Loss and Desired Weight: Longitudinal Observational Study

**DOI:** 10.2196/27153

**Published:** 2021-10-06

**Authors:** Johannes Feldhege, Markus Moessner, Stephanie Bauer

**Affiliations:** 1 Centre for Psychotherapy Research University Hospital Heidelberg Heidelberg Germany

**Keywords:** pro-eating disorder communities, weight loss, body weight, social media, linear growth models, eating disorders, pro-ED, Reddit

## Abstract

**Background:**

Online pro–eating disorder (pro-ED) communities are considered harmful because of their detrimental effects on their users’ body dissatisfaction, dieting, and help seeking. To date, it is unknown to which extent participation in pro-ED communities affects users’ body weight and desired weight loss.

**Objective:**

This study aims to investigate the changes in the current and desired body weight of users of a pro-ED community (r/proed) on the social media website Reddit over time.

**Methods:**

Data on 1170 users and the unsolicited weight information they shared with the pro-ED community were collected over a period of 15 months. Linear growth models were used to model changes in the users’ current and desired BMI over time.

**Results:**

Both current and desired BMI decreased over time, with a predicted rate of 0.087 and 0.015 BMI points per week, respectively. Weight loss was moderated by the users’ activity level in the community, with more active users losing more weight. Users with a higher baseline BMI experienced greater weight loss, but even users with a very low baseline weight (BMI <17 kg/m^2^) lost weight during their participation. In addition, users decreased their desired weight over time, with many pursuing extremely low, unrealistic weight goals. Changes in the desired weight were moderated by the baseline current BMI and baseline desired BMI. Users with higher desired weight and lower body weight at baseline decreased their desired weight more over time.

**Conclusions:**

This is the first study to demonstrate the detrimental effects of pro-ED communities in a longitudinal study based on a large data set of user-generated online data. The results extend the literature detailing the harmful effects of online pro-ED communities by showing users’ weight loss, decreases in desired weight, and that higher activity levels lead to greater weight loss. Users could be driven to pursue very low, unrealistic weight loss goals by images of very thin bodies presented in these communities.

## Introduction

### Background

Eating disorders (EDs) are severe mental disorders. Many individuals with EDs strive to lose weight to achieve a *thin ideal,* that is, an ideally slim body type portrayed as attractive in the media. Low body weight and marked weight loss contribute to the emergence of physical conditions and elevated mortality rates observed in individuals with ED [1-3].

Online pro-ED communities, also called pro-ana and pro-mia communities for anorexia nervosa and bulimia nervosa, respectively, are widespread over the internet and attract a large number of vulnerable or impaired users. These communities exist on social media platforms [4] and as self-hosted websites and blogs [5]. They have been identified as harmful to their users: participation leads to increases in ED behaviors while discouraging help seeking and recovery, thereby prolonging the duration of the mental disorder [6,7]. This has led to the ban or removal of pro-ED content and communities from social media platforms [8,9] and, in at least one instance, to the passing of a law that fines websites for hosting content that promotes excessive thinness and dieting [10]. Positive impacts of participating in these communities, such as receiving social support from similar others and feeling less alone with a stigmatized condition, have also been noted [11,12]. However, it has been suggested that this support is conditional on following unhealthy group norms and that it reinforces ED thoughts and behaviors [7,13,14].

One of the central mechanisms through which ED behaviors are promoted in these communities is *tips and tricks* for extreme weight loss, which often include behaviors such as the use of laxatives or diet pills, purging, fasting, and extreme dietary or exercise regimes [15-17]. This is potentially harmful, as adolescents report learning about new weight loss methods on pro-ED websites and using them afterward [5,18]. In addition, exposure to pro-ED websites is associated with increases in dieting [19].

But literature on the body weight and weight loss of users of pro-ED communities is scarce. Users show a variety of different body weights, with the majority having a weight in the healthy weight category as defined by the BMI classification of the World Health Organization (BMI: weight in kg/height in m^2^; underweight BMI: <18.5 kg/m^2^, healthy weight BMI: 18.5-24.99 kg/m^2^, overweight BMI: 25.0-29.99 kg/m^2^, and obese BMI ≥30 kg/m^2^ [20]) as well as sizable groups that have body weights in the underweight, overweight, and obese ranges [5]. Body dissatisfaction and concerns about body shape and weight tend to be high in users of pro-ED websites, which could lead them to pay close attention to their current weight and weight changes [12,19]. Many users of pro-ED communities use apps and web-based tools to track their body weight and food intake [21-23]. Pro-ED communities provide similar functions as weight tracking apps with *check-ins* or *status updates*, allowing users to share personal information, such as their current body weight, height, age, or gender, with the online community [4,12,24].

Individuals with EDs report lower desired weights than individuals without EDs [25]. Desired weights lower than the current weight or lower than the recommended healthy weight are associated with unhealthy behaviors in adolescents [26] and elevated ED severity in individuals with ED [27-29]. Specific features of pro-ED communities could encourage their users to lower their desired weight. They often compare themselves with other users in terms of weight and compete to be as thin as possible [7]. Another common feature of pro-ED communities, *thinspiration* content, for example, images of unrealistically thin female body shapes, is another source for comparison [15]. Exposure to thinspiration and physical appearance comparisons are positively related to ED symptoms in individuals with ED [30].

An individual’s desired weight can change over time [31,32]. Frequent changes are associated with a number of strategies to control weight and increases in physical activity [33]. Users could be encouraged to adopt lower and lower desired weights during their participation in pro-ED communities to align themselves more closely with the propagated thin ideal.

The level of user activity in a pro-ED community is positively associated with disordered eating and ED-related impairment [5]. In weight loss communities, higher levels of activity are related to greater weight loss over time [34-36].

### Objectives

Although the detrimental effects of pro-ED communities are frequently emphasized, studies demonstrating these effects longitudinally for users of a pro-ED community are lacking. This study aims at investigating the effects by modeling weight loss and changes in desired weight over the course of participation. In addition, although some studies have noted the existence of voluntarily shared weight information in pro-ED communities, the majority of studies used surveys or laboratory experiments to assess data outside of online communities. To our knowledge, this is the first study that uses data from a pro-ED community to calculate BMI and desired BMI and model their changes longitudinally.

In this study, we explored the pro-ED community r/proed on the social media website Reddit [37]. Reddit consists of thousands of communities called subreddits dedicated to topics such as sports, music, movies, news, politics, or videogames as well as physical health and mental disorders. Reddit users can write posts or comment on other users’ posts in these communities. The r/proed community, similar to other pro-ED communities, featured tips and tricks such as circumventing minimums for weight and daily intake in a fitness tracker app [23], thinspiration, and discussions about topics such as low-calorie foods or binge eating as well as social support and treatment [17]. Besides being one of the largest and most active communities related to EDs on Reddit [17], r/proed also had one feature that was relevant for this study—users could display a text field called *flair* next to their username on all their posts and comments in r/proed. The flair (the term is used by Reddit users for both the singular and plural forms) contained self-reported height, current body weight, desired weight, age, and gender. It was possible for users to create, change, or update their flair with new information at any time, thereby allowing us to estimate changes in reported weight values over time. The r/proed community was banned on November 15, 2018, by Reddit’s administrators for violating the website’s content policy, namely, for *posting of content that encourages physical harm* [38].

We hypothesize that participation in r/proed is associated with weight loss. In addition, we hypothesize that users set lower desired weights over time to align themselves more closely with the propagated thin ideal. Furthermore, we hypothesize that the level of activity in the r/proed is associated with the changes in BMI and desired BMI of the users. Highly active users are expected to lose more weight and decrease their desired BMI compared with less active users.

## Methods

### Data Collection

Data from the r/proed community and its users were collected over a period of 15 months between August 15, 2017, and November 14, 2018, until its ban. We assessed current and desired BMI using the values for height and current or desired weight at the time they appeared in the users’ flair in r/proed. The values in the flair had to be coded manually, as they could not be assigned automatically to one of the weight categories because of ambiguity of the category to which they belonged. In addition, the units for height and weight measurements were omitted from some flair requiring educated guesses to determine which measurement system was used. Three independent raters manually coded a third of the flair texts for height, body weight, and desired weight. All 3 raters also coded a common set of 322 flair texts to assess interrater reliability for height, current weight, and desired weight. The intraclass correlation coefficients (ICCs) were 1, 0.98, and 0.98. The variable representing activity levels was calculated as the average number of posts and comments a user writes in the pro-ED community per week. After rating, all values for height and weight were converted to meters and kilograms, respectively.

As the data accessed through the official Reddit application programming interface for this study were publicly available, no application for ethics approval was considered necessary. We do not report any personally identifying information in this paper to protect the privacy of all users.

### Data Analysis

We estimated separate linear growth models for the current and desired weights with individual measurement time points nested in users. The first available values for the current and desired BMIs of each user were treated as their respective baseline values. The variable time was calculated as weeks between the baseline and the respective time points. The current and desired BMIs were person centered around their respective baseline values. Baseline BMI, baseline desired BMI, and activity levels were grand mean centered and standardized. All linear growth models were estimated with maximum likelihood estimation and the Nelder-Mead optimizer using the R package lme4 [39].

We estimated four models for the current BMI by iteratively adding predictors in each model. The first model was an intercept-only model with random effects from users. This model was used to calculate ICCs to determine the amount of variance due to between-person differences. In the second model, a random slope and the fixed effect for time were added. In the third and fourth models, we added interactions of time with standardized baseline BMI and activity levels, respectively.

A similar procedure was adopted for the desired BMI values. The first model was an intercept-only model, whereas in the second model, time was added. The three following models added the interaction between time and the standardized baseline desired BMI, standardized first current BMI, and the activity level variable.

Interaction effects in the linear growth models were illustrated by simulations using the R package merTools. The graphs of the simulation results are provided.

## Results

### Descriptive Statistics

A total of 16,241 different flair texts were created by 5372 users during the data collection period. Of the 5372 users, 4101 had to be excluded from the analysis because they had only one measurement point for the current and desired weight. We performed sanity checks of the coded data and excluded 13 users because they had unrealistic values for height, current, or desired weight, such as a goal weight of 0 or a current weight BMI of 100, or because their flair showed implausible changes over time, such as a height increase of 5 cm in 1 day. The final data set consisted of 1170 users with 5193 flair texts. The descriptive statistics of the sample are presented in Table 1. At baseline, the average BMI was 22.32 (SD 4.08), whereas the average desired BMI was 18.55 (SD 2.33). Users participated in the community for an average of 25.00 weeks (SD 19.36) during our data collection. Overall, 1.2% (14/1170) users participated from the beginning of data collection to the last week before the community was banned, and 50% (585/1170) users participated for more than 20 weeks.

**Table 1:** Demographics, participation characteristics, and baseline values for the current and desired weight of users of an eating disorder community on Reddit (n=1170).

				Values
**Demographics**				
	**Gender^a^, n (%)**			
		Female	891 (76.15)	
		Male	63 (5.38)	
		Nonbinary	25 (2.14)	
		Transgender	9 (0.77)	
	**Age^b^ (years)**			
		Value, mean (SD)	22.54 (4.68)	
		Value, median (range)	22 (13.60-59.00)	
**Participation characteristics**				
	**Number of flair texts**			
		Value, mean (SD)	4.44 (3.93)	
		Value, median (range)	3 (2-62)	
	**Time^c^**			
		Value, mean (SD)	13.27 (14.67)	
		Value, median (range)	7.59 (0.00-64.68)	
	**Activity level^d^**			
		Value, mean (SD)	7.68 (29.85)	
		Value, median (range)	2.75 (0.0-759.32)	
**Baseline values**				
	**Height (cm)**			
		Value, mean (SD)	164.91 (7.72)	
		Value, median (range)	165.10 (142.24-195.58)	
	**Current weight (kg)**			
		Value, mean (SD)	60.80 (12.42)	
		Value, median (range)	58.29 (34.47-135.35)	
	**Current BMI (kg/m^2^)^e^**			
		Value, mean (SD)	22.32 (4.08)	
		Value, median (range)	21.48 (14.20-46.74)	
	**Desired BMI (kg/m^2^)^f^**			
		Value, mean (SD)	18.55 (2.33)	
		Value, median (range)	18.31 (12.02-42.29)	

^a^Overall, 15.56% (182/1170) of users did not report their gender.

^b^Overall, 50.85% (595/1170) of users did not report their age.

^c^Weeks between the first and last flair of a user.

^d^Number of posts and comments of a user per week.

^e^Current BMI = (current weight in kg)/(height in m^2^).

^f^Desired BMI = (desired weight in kg)/(height in m^2^).

### Linear Growth Models for the Current BMI

The ICC for BMI, calculated using the intercept-only model, was 0.38, indicating considerable variance between users. Compared with the intercept-only model, the three subsequent models, each adding one additional predictor, showed marked improvements in the goodness-of-fit measures Akaike information criterion (AIC) and Bayesian information criterion (BIC). The fourth model had the lowest AIC and BIC values and thus was chosen as the final model (results for all four models are provided in Table 2).

**Figure 1:**
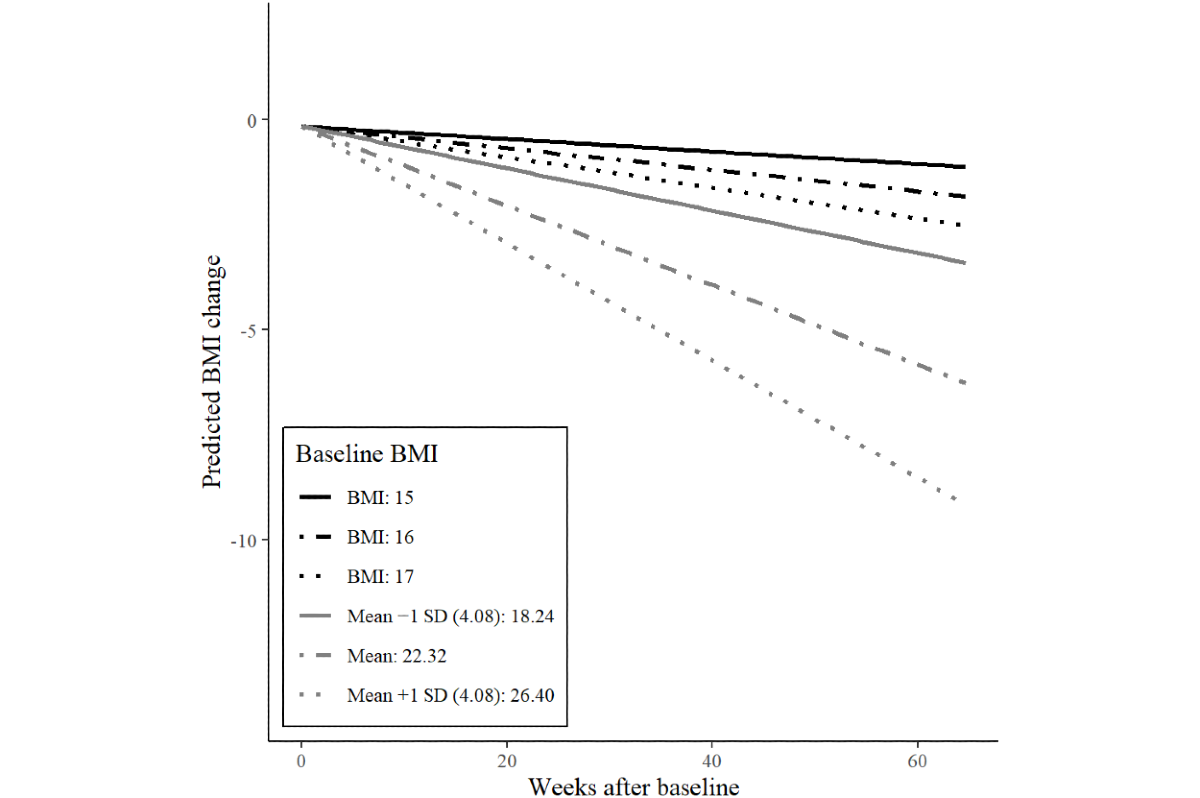
Interaction effects of time and baseline current BMI on current weight BMI change of 1170 users of an eating disorder community on Reddit.

The final model shows that users lost weight over time, with a predicted rate of 0.087 BMI points per week. Users with a higher baseline BMI and higher activity levels experienced greater weight loss. The interaction between time and baseline BMI on weight loss is shown in Figure 1. It shows that even users with low BMIs at baseline (BMIs between 15 and 18.24) lost some weight during their participation in the pro-ED community.

**Table 2:** Linear growth models for the current BMI of 1170 users of an eating disorder community on Reddit.

		Model 1	Model 2	Model 3	Model 4
**Fixed effect estimates (SE)**					
	Intercept	−0.473 (0.025)	−0.165 (0.013)	−0.169 (0.013)	−0.165 (0.013)
	Time	—^a^	−0.074 (0.004)	−0.072 (0.004)	−0.087 (0.005)
	Time×baseline current BMI interaction^b^	—	—	−0.044 (0.004)	−0.043 (0.004)
	Time×activity interaction^b^	—	—	—	−0.118 (0.020)
**Random effects variance**					
	Intercept	0.512	0.068	0.068	0.069
	Residual	0.829	0.250	0.251	0.250
	Slope	—	0.011	0.009	0.009
**Goodness-of-fit measures**					
	Akaike information criterion	15,172.918	10,504.541	10,387.654	10,353.948
	Bayesian information criterion	15,192.584	10,543.871	10,433.540	10,406.389
	Log likelihood	−7583.459	−5246.271	−5186.827	−5168.974

^a^—: not applicable.

^b^Main effects of baseline current BMI and activity were excluded from their models as their inclusion lowered the model fit*.*

**Figure 2:**
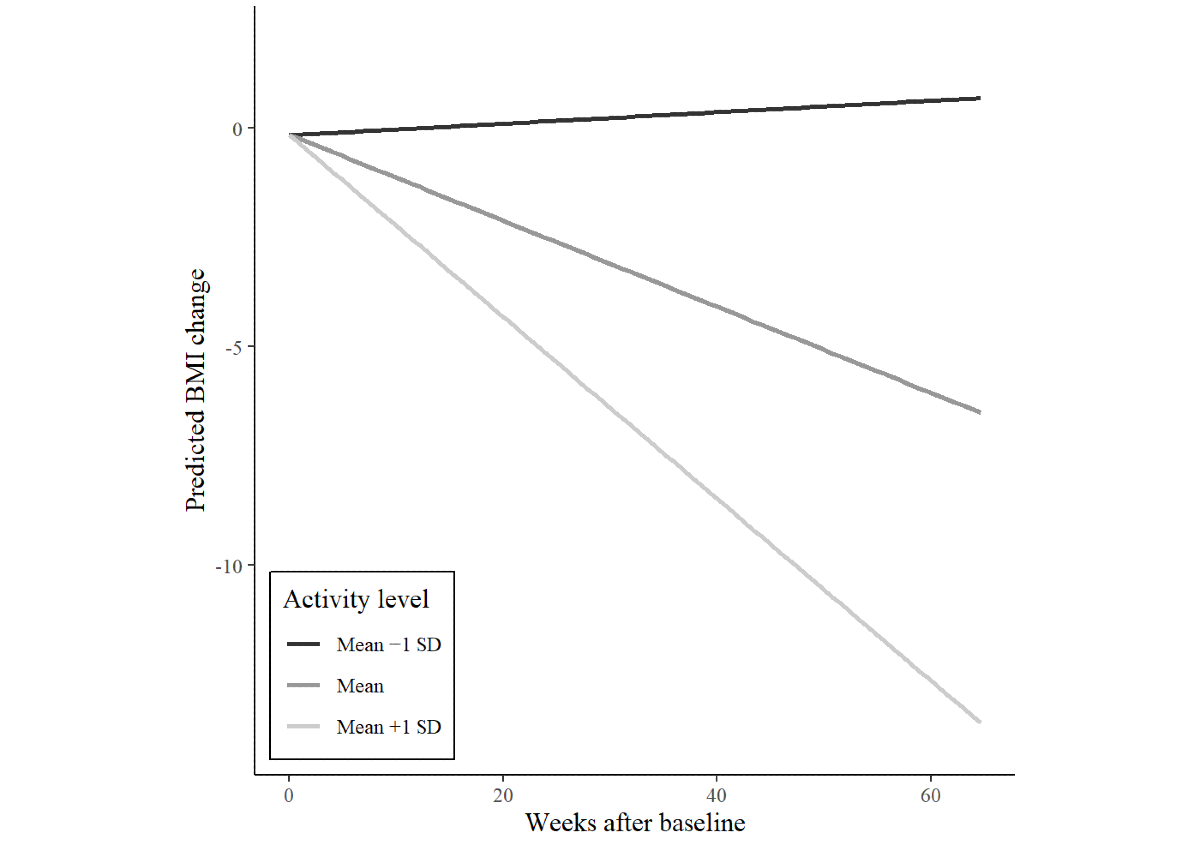
Interaction effects of time and activity level on current weight BMI change of 1170 users of an eating disorder community on Reddit.

Figure 2 shows the interaction between the activity level and time on weight loss. Although greater activity levels were associated with greater weight loss for users at mean or above mean activity levels, users with low activity levels were predicted to gain weight.

### Linear Growth Models for the Desired BMI

The intercept-only model for the desired BMI has an ICC of 0.41, which indicates substantial variance between users. The three subsequent models showed marked improvements in the AIC and BIC fit measures. Including the interaction of activity and the time variable (model 5) does not improve the model fit. Therefore, the fourth model was considered as the final model (Table 3).

**Figure 3:**
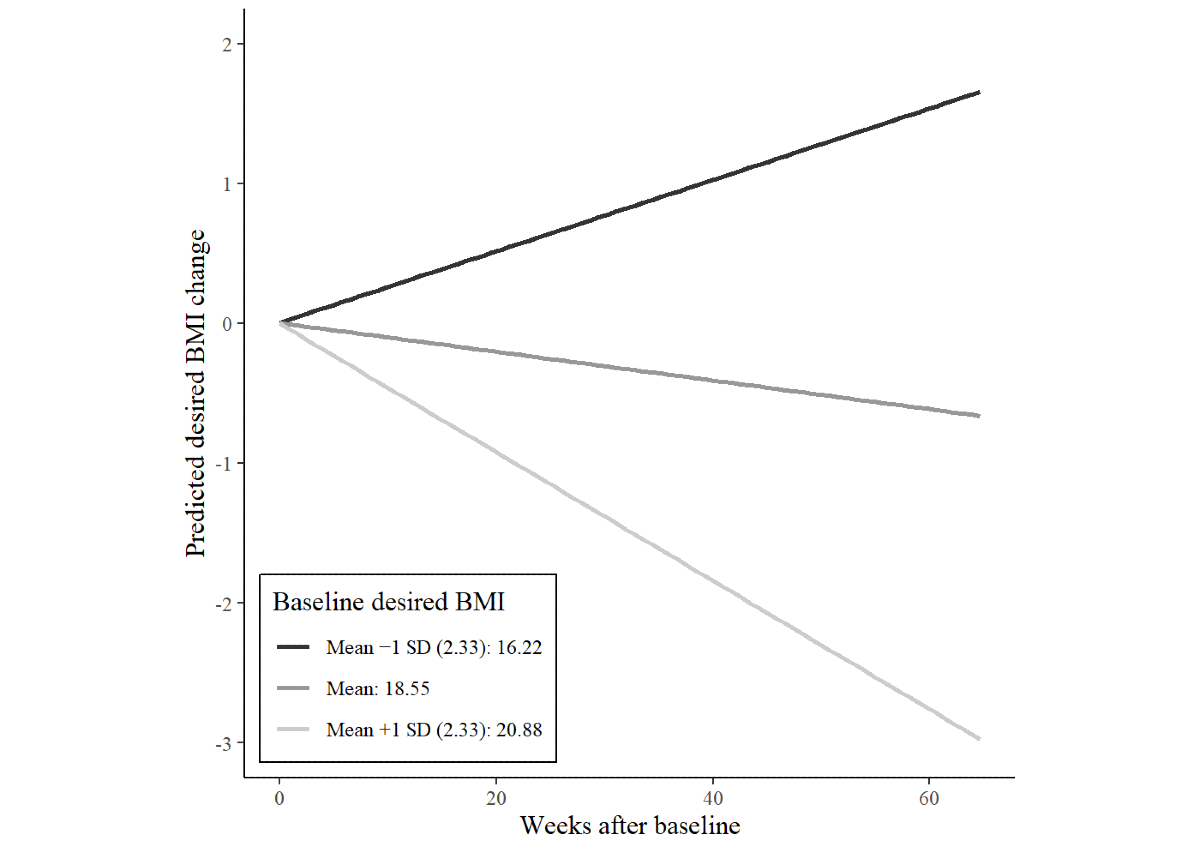
Interaction effects of time and baseline desired BMI on desired weight BMI change of 1170 users of an eating disorder community on Reddit.

On average, users’ desired weight decreased by 0.014 BMI points per week. Those with average or above average baseline desired BMIs reduced their desired weight, whereas those with below average baseline desired BMI increased their desired BMI over time. Figure 3 illustrates the interactions of time and baseline desired BMI on desired BMI graphically.

**Table 3:** Linear growth models for the desired BMI of 1170 users of an eating disorder community on Reddit.

		Model 1	Model 2	Model 3	Model 4	Model 5
**Fixed effect estimates (SE)**						
	Intercept	−0.075 (0.021)	−0.003 (0.01)	0.002 (0.017)	0.002 (0.017)	0.003 (0.017)
	Time	—^a^	−0.015 (0.003)	−0.015 (0.003)	−0.014 (0.003)	−0.015 (0.003)
	Time×baseline desired BMI interaction^b^	—	—	−0.017 (0.003)	−0.033 (0.003)	−0.033 (0.003)
	Time×baseline current BMI interaction^b^	—	—	—	0.026 (0.003)	0.026 (0.003)
	Time×activity interaction^b^	—	—	—	—	−0.010 (0.015)
**Random effects variance**						
	Intercept	0.379	0.185	0.187	0.187	0.187
	Residual	0.534	0.335	0.336	0.335	0.335
	Slope	—	0.004	0.004	0.003	0.003
**Goodness-of-fit measures**						
	Akaike information criterion	12999.600	11627.828	11593.609	11538.863	11540.415
	Bayesian information criterion	13019.265	11667.159	11639.494	11591.304	11599.411
	Log likelihood	−6496.800	−5807.914	−5789.804	−5761.432	−5761.208

^a^—: not applicable.

^b^Main effects of baseline desired BMI, baseline current BMI, and activity were excluded from their models as their inclusion lowered the model fit.

**Figure 4:**
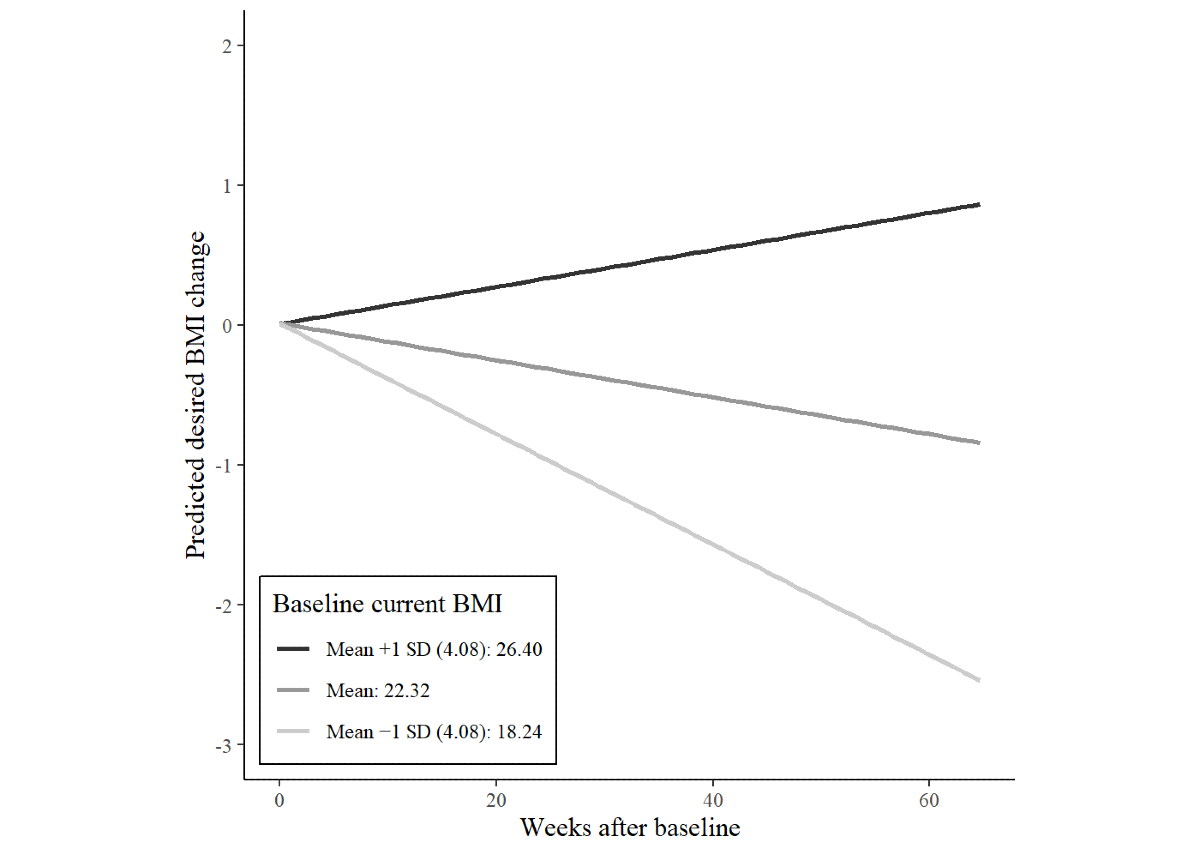
Interaction effects of time and baseline current BMI on desired weight BMI change of 1170 users of an eating disorder community on Reddit.

The interaction between baseline current BMI and time on desired BMI was positive, indicating that a higher baseline current BMI was associated with a slower decrease or even an increase in desired BMI over time. Figure 4 illustrates this interaction.

## Discussion

### Principal Findings

This study is the first to investigate changes in the current and desired BMI over a period of 15 months using user-generated data from an online pro-ED community. We were able to demonstrate sustained decreases in the BMI of r/proed users over the course of their participation. Although baseline BMI moderated the effect of time, even participants with very low baseline BMIs of 15-17 kg/m^2^ still lost weight. The fact that even users with very low baseline BMIs lost weight over the course of their participation is alarming, as this exacerbates the risks associated with low body weight. However, weight loss is also related to clinical complications in individuals with body weight above the underweight category [3]. In addition, the level of activity in the r/proed community was associated with weight loss. The more active users contributed to the pro-ED community, the more weight they lost over time (Figure 2). In addition to evidence that more active users experience greater ED-related impairment [5], our results suggest that they are also at greater risk of unhealthy weight loss over the course of their participation. Baseline BMI as a moderator of weight loss over time is consistent with results from a case study on a weight loss community on Reddit [36]. Those at higher baseline BMIs might have been more motivated to lose weight by a perception of excess weight in a community that puts a high value on the thin ideal [36]. In addition to decreases in BMI, we could also demonstrate decreases in users’ desired BMIs over the course of their participation. This suggests that users did not have one fixed weight goal but that ever-decreasing weight goals stayed out of their reach, even when they were losing weight. Baseline BMI moderated changes in the desired BMI (Figure 4). Users with higher baseline BMI increased their desired BMI from initially more unrealistic to healthier values over time. However, users with average or below average baseline BMI decreased their desired BMI over time. This is concerning, as these users are very likely to experience high body weight dissatisfaction although they already had low or underweight body weights.

The interaction of baseline desired BMI and time (Figure 3) demonstrates that the desired BMI of the users aligns over time. In a way, the pro-ED community moves toward a common middle ground of desired weight. Such a concurrence in the level of desired BMI could be because of posts of thinspiration images featuring women of similar, thin body types or because of users comparing and adjusting to the desired weight in the flair of other users.

In summary, the results strongly support the detrimental effects of participating in online pro-ED communities and point to the risks associated with these communities. In view of these risks, potential benefits such as receiving social support and reducing loneliness appear negligible [11].

Users’ average desired BMI at baseline was just above the cutoff for underweight and thus at a similar level to that of a sample of women with ED [25] but considerably lower than that of a population sample [32]. In this study, the sample’s average baseline desired weight was 20% lower than the average baseline current weight and thus higher than the recommended realistic weight loss goals of 5%-10% below the actual body weight [40]. Users pursuing unrealistic weight loss goals that extend into the underweight range point to the influence of thinspiration and social comparisons on weight goal setting. A lower desired weight in relation to actual body weight has also been linked to greater severity of ED [27,28].

We could not identify the specific processes and factors that caused these effects and yielded weight loss or decrease in the desired BMI. Cross-sectional and experimental studies have shown that exposure to pro-ED communities is linked to increased dieting and body dissatisfaction; however, it is unclear whether these are enduring effects [19]. In addition, online pro-ED communities are considered harmful because they decrease the likelihood of seeking help and lead to increased ED symptoms [7,19,41]. Moreover, encouragement and social reinforcement from other users may foster extreme weight loss techniques [11].

### Strengths and Limitations

Our analyses show that a high number of users used features such as flair to self-report their height, current, and desired weight in the pro-ED community and that it is possible to reliably extract meaningful information on ED impairment from online ED communities. Although self-reports of weight and height can be inaccurate, users of pro-ED communities, young women and women with EDs, tend to report their height and weight more accurately [42]. The results of this study apply only to active users in the pro-ED community. It remains unclear whether the findings can be generalized to lurkers who do not actively contribute to the community. In addition, we could not determine whether individuals were active with more than one Reddit account during the period of our study, either using multiple accounts simultaneously or sequentially. Another limitation of the study is that its observational study design does not allow to conclude causality. However, conducting randomized controlled trials to investigate the detrimental effects of potentially harmful online communities longitudinally is not feasible. The users’ activity on other social media platforms besides Reddit, such as Facebook or Instagram, was not assessed in this study. These platforms can also have detrimental effects on ED symptomatology [43,44]. However, the moderating effects of the activity level support the validity of the results. Although changes in BMI and desired BMI can be considered valid proxies for ED impairment in this population (especially for the subgroup of users with very low BMIs), they represent only one aspect of ED-related impairment, and more comprehensive assessments of ED impairment would be beneficial.

### Conclusions

In November 2018, the administrators of Reddit issued a ban on the r/proed community. Although the subject of this study no longer exists, we consider its results to be an important addition to the literature. They can inform our understanding of other newly created or existing communities with similar processes and effects to those in r/proed. After the ban of r/proed, almost immediately, new communities appeared in its place, with most of them being quickly taken down by Reddit’s administrators. One such community, r/EDAnonymous [45], has avoided a ban so far, possibly because of its stricter rules that do not allow weight and height measurements in flair. This is unlikely to prevent or stop detrimental effects, such as weight loss and decreases in desired weight, as we showed in this study, instead rendering these processes invisible to social media admins and researchers. Uncovering these processes was only possible by studying the now-banned r/proed.

In general, it seems unlikely that it will be possible to ban pro-ED communities completely from the internet or even from major social media platforms, such as Pinterest, Instagram, and Tumblr [46]. Therefore, it is of utmost importance to learn more about the detrimental effects of these communities, their social structures, the processes that take place, and the factors mediating the effects of web-based communication on ED symptomatology or mental health in general. Future research should focus on identifying the mechanisms that occur within online communities. Observational studies, such as this study, have to complement cross-sectional and experimental research to gain a deeper understanding of the processes and social structures in these communities and to counteract potentially detrimental effects on their users. The deeper understanding gained from this study and other similar research is also relevant for clinical work. Therapists should discuss social media habits and potentially harmful social media use with their clients. Therapeutic and preventive interventions for EDs, especially those for adolescents and emerging adults, should work toward healthier use of social media, that is, by teaching social media literacy [47].

## Abbreviations

AIC: Akaike information criterion
BIC: Bayesian information criterion
ED: eating disorder
ICC: intraclass correlation coefficient
